# Incisions in Hepatobiliopancreatic Surgery: Surgical Anatomy and its Influence to Open and Close the Abdomen

**DOI:** 10.3389/jaws.2023.11123

**Published:** 2023-03-22

**Authors:** Manuel Medina Pedrique, Álvaro Robin Valle de Lersundi, Adriana Avilés Oliveros, Sara Morejón Ruiz, Javier López-Monclús, Joaquín Munoz-Rodriguez, Luis Alberto Blázquez Hernando, Javier Martinez Caballero, Miguel Ángel García-Urena

**Affiliations:** ^1^ Grupo de Investigación de Pared Abdominal Compleja, Hospital Universitario del Henares, Facultad de Ciencias de la Salud, Universidad Francisco de Vitoria, Madrid, Spain; ^2^ General and Digestive Surgery Department, Hospital Universitario Puerta de Hierro, Autónoma University of Madrid, Madrid, Spain; ^3^ General and Digestive Surgery Department, Hospital Universitario Ramón y Cajal, Alcalá de Henares University Madrid, Madrid, Spain

**Keywords:** incisional hernia, incisional hernia prevention, hepatobiliary surgery, mesh prevention, subcostal incisions

## Abstract

Incisions performed for hepato-pancreatic-biliary (HPB) surgery are diverse, and can be a challenge both to perform correctly as well as to be properly closed. The anatomy of the region overlaps muscular layers and has a rich vascular and nervous supply. These structures are fundamental for the correct functionality of the abdominal wall. When performing certain types of incisions, damage to the muscular or neurovascular component of the abdominal wall, as well as an inadequate closure technique may influence in the development of long-term complications as incisional hernias (IH) or bulging. Considering that both may impair quality of life and that are complex to repair, prevention becomes essential during these procedures. With the currently available evidence, there is no clear recommendation on which is the better incision or what is the best method of closure. Despite the lack of sufficient data, the following review aims to correlate the anatomical knowledge learned from posterior component separation with the incisions performed in hepato-pancreatic-biliary (HPB) surgery and their consequences on incisional hernia formation. Overall, there is data that suggests some key points to perform these incisions: avoid vertical components and very lateral extensions, subcostal should be incised at least 2 cm from costal margin, multilayered suturing using small bites technique and consider the use of a prophylactic mesh in high-risk patients. Nevertheless, the lack of evidence prevents from the possibility of making any strong recommendations.

## Introduction

Incisional hernias (IH) consist of any abdominal defect in the vicinity of a postoperative scar that can be detected by clinical examination or by radiographic studies (1). There is no doubt that incisional hernias are an important public health issue, due to an estimated incidence of up to 37% (1), and also due to the implications they have on the patient’s quality of life. The patient’s symptoms may include pain, limitation of daily life activities, skin problems due to ulceration/infection, incarceration, and other complaints that may require an elective or even emergent surgical procedure (2).

Although it has been indicated that IH seems to be more frequent after midline incisions than off-midline wounds (3), hernias after hepato-bilio-pancreatic (HPB) surgery using subcostal or transverse incisions are considered complex and their subsequent repair may be a challenging procedure (4–6). HPB surgeries may also require a combination of midline and lateral incisions that may make it even more difficult to repair (7, 8). The combination of the different type of incisions made in each patient and the scarcity of the evidence available hinders any attempt to establish which is the best incision to avoid herniation in HPB surgery, or what can we do to decrease the risk of IH formation. The increasing anatomical knowledge from the applications of component separation techniques that can be used to treat IH after HPB (9, 10), has also suggested us to critically evaluate the potential damage to anatomical structures in HPB surgery and its influence in the morphology and the function of the abdominal wall. The purpose of this narrative review is to provide an updated analysis on the current HPB incisions from an anatomical perspective in order to raise awareness among HPB surgeons of the potential influence of their different incisions and their closures on the development of IH.

## Surgical Anatomy

**FIGURE 1:**
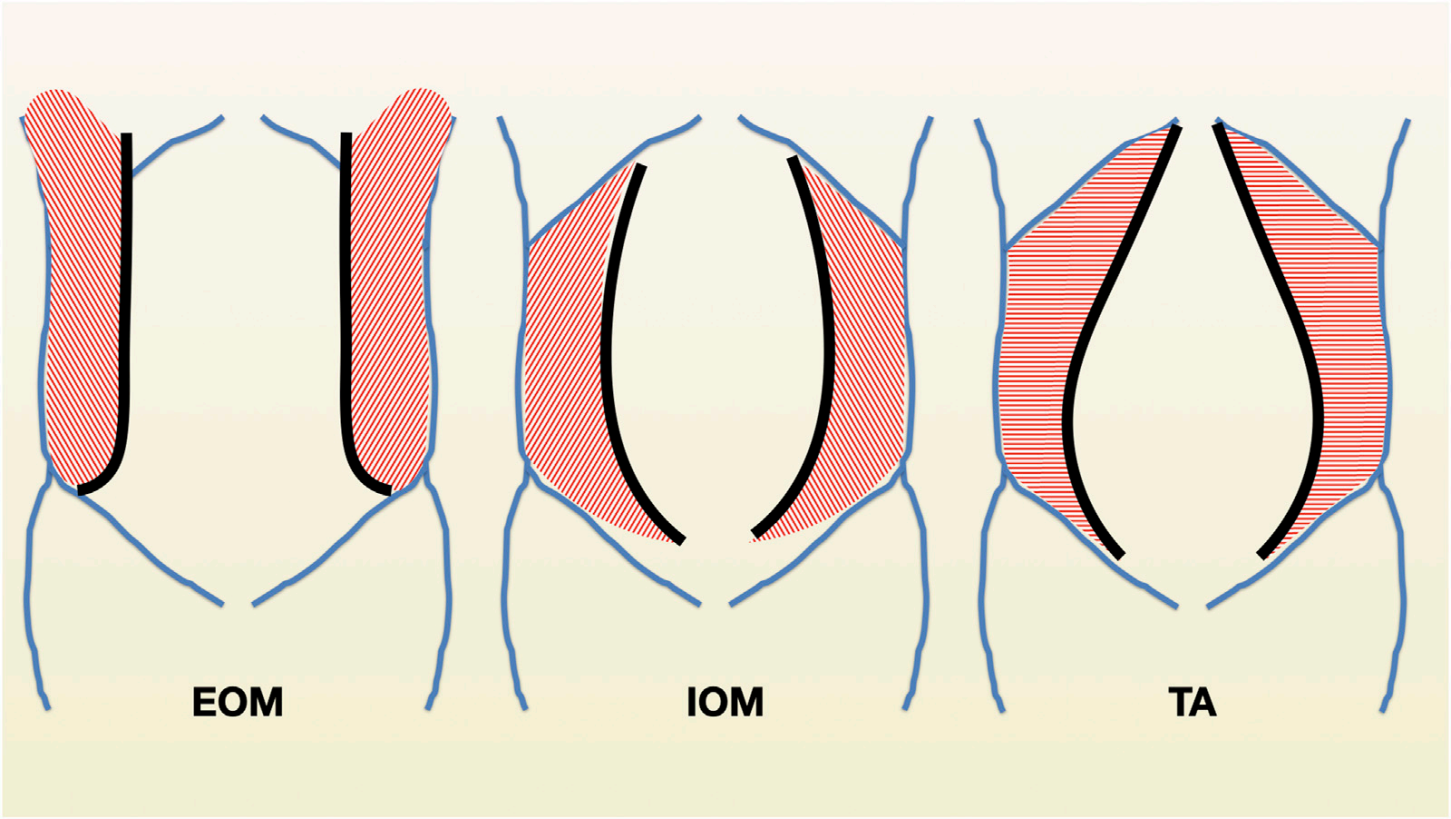
Schematic representation of the myoaponeurotic limits of the external oblique muscles (EOM), internal oblique muscle (IOM) and transversus abdominis (TA).

The anterior abdominal wall has been classically described as and hexagonal area that is limited by the xiphoid process and the costal margin superiorly, the pubic bone and the inguinal ligaments inferiorly, and has a lateral extension back to the quadratus lumborum and the erector spinae muscle (11, 12). Within these limits, various muscular groups overlap and give functionality to the abdominal wall. The muscles have been also divided into midline group: rectus abdominis (RM) and the pyramidalis muscle, and anterolateral group: external oblique muscle (EOM), internal oblique muscle (IOM) and transversus abdominis muscle (TA). The RM is the main component of the midline muscle group. Both muscles originate from the pubic crest and go from bottom to top to insert in the xiphoid process and the anterior surface of the 5th–7th costal cartilages (12). In the superior hemiabdomen, the RM has a slightly oblique direction towards lateral and is enveloped between the anterior and posterior rectus sheaths. The main component of the anterior rectus sheath is the aponeurotic insertion of the EOM on linea alba. The understanding of the myoaponeurotic limit of the EOM may help to better approximate the borders incised when closing this layer (Figure 1). The IOM fibers run perpendicular to those of the EOM and their aponeurotic insertion divides into anterior and posterior lamellas. The anterior lamella fuses the aponeurotic insertion of the EOM forming the anterior rectus sheath. The posterior lamella of the IOM contributes to the posterior rectus sheath. In the superior hemiabdomen, the posterior rectus sheath is made of this posterior lamella and the TA. The fibers of TA run horizontally and almost reach the midline in the epigastric area. The myoaponeurotic limit of the TA muscle is called linea semilunaris. While the space between EOM and IOM can be easily dissected without injuring any vascular or neural structures, the space between TA and IO muscle is quite difficult to dissect and the branches of the intercostal nerves run along this space. While outside the linea semilunaris, the space between the peritoneum and TA muscle can be effortlessly developed due to the abundant preperitoneal fat (13), medial to the linea semilunaris the peritoneum is really attached to the posterior rectus sheath and cannot be separated independently.

Knowledge of the anatomy is crucial when performing incisions or closing them in the upper abdominal wall, since entering the abdomen *via* a Kocher or a Chevron incision implies division of several muscles and in some cases of the vascular and nervous supplies that give functionality to the abdominal wall. Therefore, the two main long-term consequences after these lateral incisions are: hernias, muscle denervation (14, 15). Incisional hernia happens when there is a defect of the musculoaponeurotic layers of the abdominal wall, which responds to several risk factors similar to incisional hernia formation in other locations (p.e: obesity, surgical site infection, inadequate closure technique or impaired wound healing). Bulging occurs as a consequence of injury to the nerves that leads to denervation and subsequent atrophy of the lateral abdominal wall muscles. The consequence of this injury translates into a bulge in the surgical scar with no real fascial defect (15). Most of times, both consequences arise together: a fascial defect with an associated muscle atrophy.

## Incisions in HPB Surgery

The main goal of any incision in surgery is to provide adequate exposure for the planned procedure while being sufficient to address any change due to intraoperative findings or complications. Other concerns include the preservation of abdominal wall functionality as well as allowing abdominal wall healing minimizing the risk of abdominal wall disruption or a posterior hernia formation (16). The need for quick access, accounting for previous scars/cosmetic results while minimizing postoperative pain are also important factors to take into consideration.

In HPB surgery, several incisions have been described to approach the upper abdomen. We will provide a brief description of each, while also naming a few anatomical key points to take into consideration when performing or closing them.

### Midline Incision

The most commonly used incision in open surgery, the midline incision is done along the craniocaudal axis at the linea alba. Since the midline is an avascular plane, risk of nerve or muscular injury is very low (16). Although it has widespread use across all areas of surgery, it is not the most common incision used to perform HPB procedures. Chen-Xu et al found in a retrospective study that midline incision was used in HPB surgery in 16% out of 444 patients (17). Nevertheless, the midline component of some hybrid incisions used in HPB surgery is at a high risk of incisional hernia formation (6).

### Oblique Incisions

**FIGURE 2:**
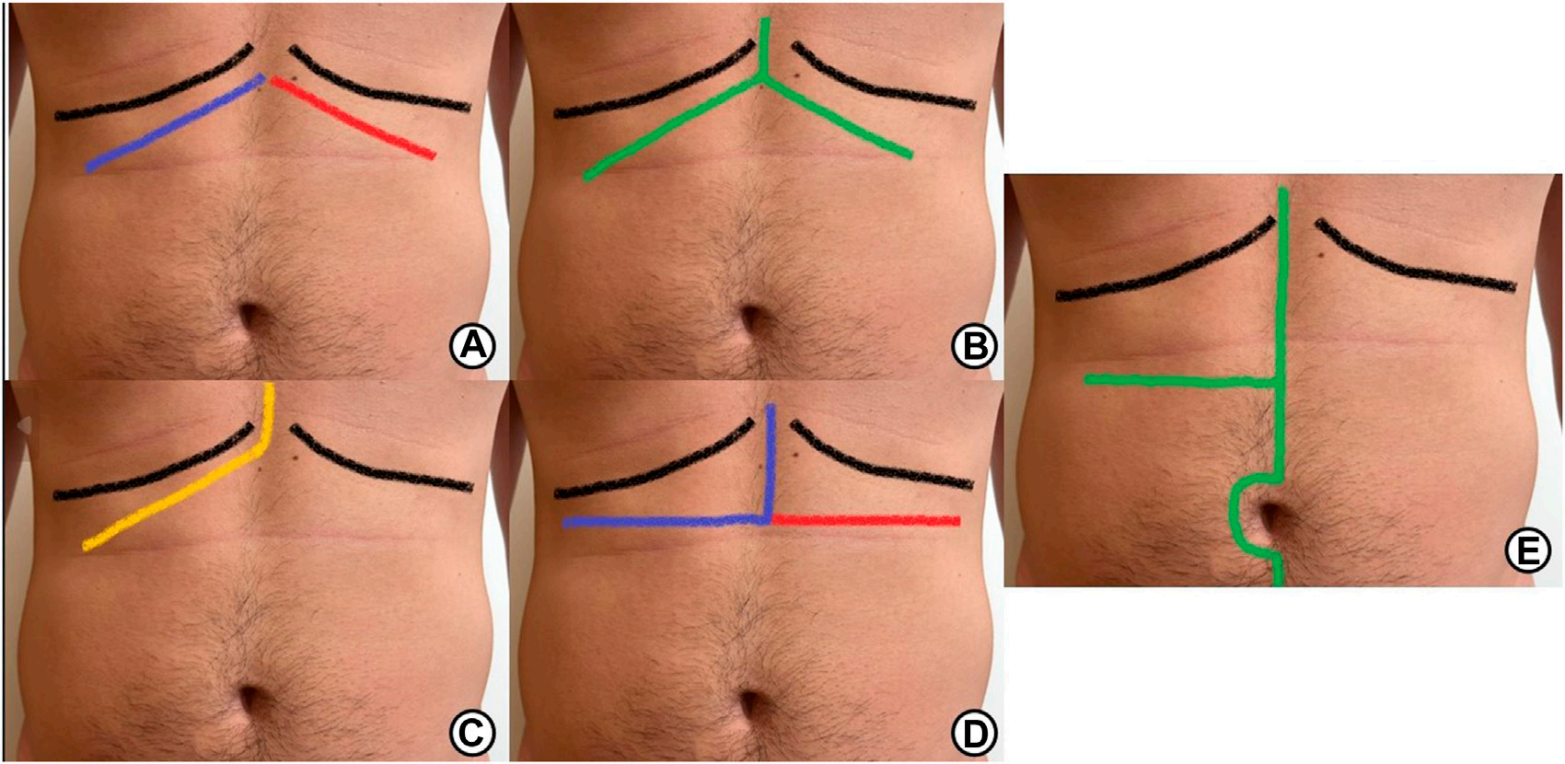
Different types of incisions used in HPB surgery: **(A)** Lines of incision of unilateral right subcostal (Kocher incision, blue line) and bilateral subcostal incision (Blue and red lines). **(B)** Lines of incision of a bilateral subcostal incision with superior vertical midline extension (Mercedes-Benz). **(C)** Line of incision of an extended subcostal right incision or J incision. **(D)** Lines of incision of a reverse L incision (blue line) and a reverse T incision (blue and red line). **(E)** Line of incision of a right transverse incision with vertical extension.

A Kocher incision is defined as a subcostal incision performed 2 cm parallel to the costal border, either at the right or left side of the abdomen (Figure 2). This incision divides the anterior rectus sheath, the RM, and the posterior rectus sheath. It requires cautery or ligation of the superior epigastric vessels which are usually divided into 2 or 3 branches along the rectus muscle (18–20). If extended laterally, the 3 lateral abdominal wall muscles are also divided. It is one of the most used incisions in HPB as it provides great exposure to hard-to-reach structures such as the suprahepatic veins, cava vein, biliary tract, pancreas, duodenum, or even the spleen. This wound can be extended to the other side, named a bilateral subcostal or **Chevron incision** (18–20)**,** and it can also extend superiorly at the midline towards the xiphoid process, called the **Mercedes-Benz incision** (Figure 2) (18–20). Despite its advantages in HPB surgery, subcostal incisions tend to produce more postoperative pain and have a less satisfactory cosmetic outcome than midline incisions (16).

**FIGURE 3:**
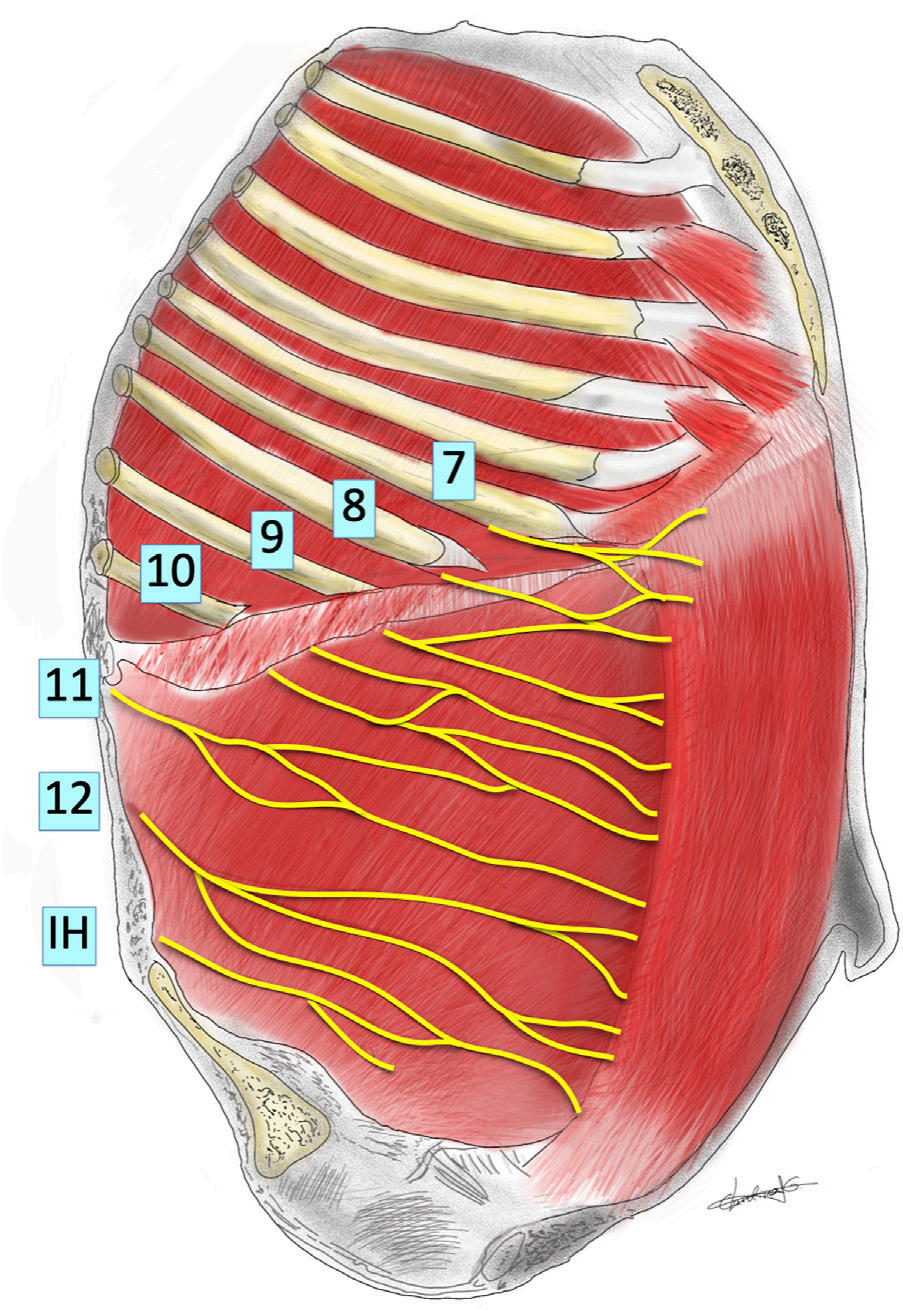
Schematic representation of innervation of abdominal wall looking into the lateral abdominal wall from inside the abdomen. The pleura, peritoneum, subcostal, intracostal and transversus abdominis muscles have been removed. The thoracic nerves (numbered) and the iliohypogastric nerve (IH) are shown, running under the inner aspect of the internal intercostal and internal oblique muscles. Based on the description of Davies and Fahim.

**FIGURE 4:**
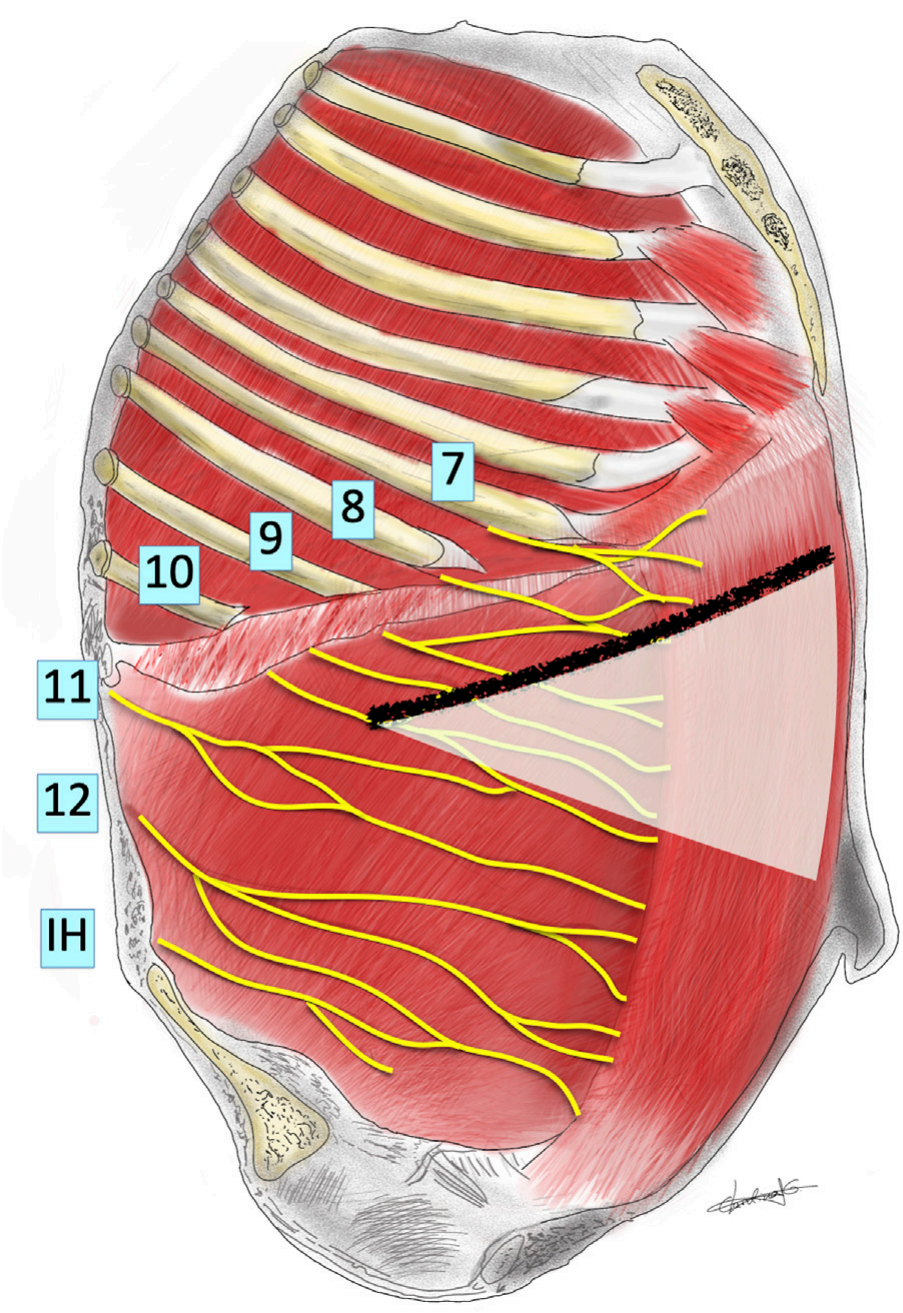
Schematic representation of innervation of abdominal wall looking into the lateral abdominal wall from inside the abdomen showing a subcostal incision and the theoretical area of denervation produced (area shaded white).

When performing these incisions, special care must be taken as muscular (RM, EOM, IOM and TA), vascular and neural structures can be injured and could have influence on the development of IH, like the intercostal nerves and the lateral border of the rectus sheaths where, as mentioned before, the lateral wall muscles insert forming the anterior and posterior rectus sheaths. According to anatomical descriptions (11, 21, 22), branches of the 7th, 8th, 9th, and probably 10th intercostal nerves must be systematically cut when performing subcostal incisions (Figures 3, 4). If the incision is extended more laterally, branches from 11th and 12th nerves could also be injured. So, the motor innervation of the supraumbilical segment of the rectus muscles is impaired to the extent of the injury of these intercostal nerves, causing ipsilateral rectus muscle and TA atrophy at the supraumbilical area (16).

Probably this type of incision almost perpendicular to the direction of the nerves may not be considered an ideal one from an anatomical point of view. The remaining EOM may also participate in the IH formation by its contraction perpendicular to the direction of the incision, while the IOM direction of fibers runs parallel to the incision (11, 21–23). From an anatomical point of view, the less we extend laterally the incision the less probability of injuring more nerves (Figure 3). When feasible, it would be advisable to extend the incision to the contralateral side better than performing a more lateral extension.

### Hybrid Incisions

These incisions are defined by the combination of both midlines/vertical and transverse laparotomies. They are usually named by the form of the incision. The most used incision of this type in HPB surgery is a J-shaped incision (Figure 2), which consists of a right subcostal incision with a medial to cranial extension to the xiphoid process. Other hybrid incisions used are the Makuuchi incision or reverse-L, a reverse-T incision, and a right transverse incision with vertical extension (Figure 2) (24).

The consequence of a transverse incision is quite similar to a subcostal, although the incision only runs parallel to the TA muscle. This incision may preserve innervation to the segment of the rectus muscle above the scar but, laterally, can injure 11th and 12th branches that are considered the most important innervation contributors to the anterior abdominal wall (2, 23).

The vertical extension of a subcostal (Kocher) or transverse incision has been traditionally considered a “hernia-formation” incision. In fact, in the recent systematic review by Davey (23), hybrid incisions seemed to develop more IH than transverse incisions, although the quality of the studies included had significant methodological weaknesses. Probably, the reason may be the addition of the lateral traction on both sides through the midline scar to an already weakened subcostal or transverse incision as previously mentioned.

Based on anatomy, recommendations on how to make these incisions can be summarized in the following.

• Establish beforehand a proper wound size for adequate exposure, trying to be as conservative as possible (18–20).
• Avoid vertical extension of a transverse incision.
• Avoid lateral extension, or disruption of the intercostal space between the 11th and 12th ribs (2).
• Distance the incision at least 2–3 cm from the costal margin. Performing the incision to close to the subcostal area might not allow to preserve sufficient fascia for closure (11, 19).
• Make sure to perform adequate hemostasis to avoid hematoma formation, especially when dividing muscle fibers (2). The superior epigastric artery divides in branches around the region of the sixth costal cartilage. It passes inferolateral and pierces the posterior rectus sheath to lie on the posterior surface of the rectus abdominis muscle. When performing subcostal incisions, this artery or its branches should be carefully controlled after dividing the rectus muscle (11, 19).
• The nerves of the anterior abdominal wall run parallel to the muscle fibers and to the vascular supply, from lateral to medial. To avoid injury, when possible, follow the path of the nerves towards the midline and try to preserve them (2).

## Incisional Hernia After HPB Surgery

### Incidence

Several studies have reported the incidence of incisional hernia after HPB surgery (25). Nilsson et al. documented an incidence of 30.5%, in which 3 incisions were reviewed: midline-only incisional hernias appeared in 84.6%, midline plus subcostal or lateral incisional hernias appeared in 10.3%, and finally subcostal only incisional hernias showed up in 3.8% (26). Togo et al. published a retrospective review of 626 patients that underwent partial hepatectomy *via* median, J-shaped, right transverse with vertical extension incisions (RTVE) and bilateral transverse incision or reverse-T. The frequency of incisional hernia for each incision was 6.3% for midline, 4.7% for J-shaped incision, 5.4% for RTVE, and 21.7% for reverse-T incision (27). It would have been interesting to detail what component of the hybrid incisions was affected the most, either the midline or the lateral component of the wound. Chen-Xu et al. described the incidence of incisional hernia after HPB surgery in a retrospective review of 696 patients. They described the frequency of incisional hernia in those patients submitted to pancreatic surgery (incidence of 10.5% at 24 months of follow-up) and those submitted to hepatobiliary surgery (incidence of 27% at 72 months of follow-up). The most performed incision in their study was the J-shaped incision (64.7%). Overall incidence was estimated at 21.6%, which is a very significant number of patients that develop this complication during their follow-up. They also studied potential risk factors for incisional hernia occurrence in these patients, detailing that for pancreatic surgery a height greater than 167.5 cm, a subcutaneous fat >23.3 mm, and wound infection/dehiscence increased frequency. In hepatobiliary surgery, risk factors identified were a BMI >26.0 kg/m2 and having a perirenal fat pad >14.7 mm (17). Both of these variables correlate with the fact that obesity is an important predisposing factor for incisional hernia development. Finally, Davey et al. in a recent systematic review pooled a total of 5,427 patients and reported an incidence for incisional hernia of 15% in those patients with hybrid incisions (J-shaped, Mercedes-Benz, reverse-L, reverse-T, and RTVE) at 42 months of follow up, compared to a pooled incidence for incisional hernia of 6% for those patients with transverse incisions with a mean follow up of 17.5 months (23).

A more recent study carried out by Lida et al. retrospectively reviewed 1,057 patients who underwent open hepatectomy *via* J-shaped, reverse L-shaped, reverse T-shaped, and Mercedes-Benz incision. They had a reported incidence of 5.9% during 3 years of follow-up, and associated risk factors for IH development were age ≥65 years, diabetes mellitus, and albumin levels <3.5 g/dL. They also differentiated which incision had a greater incidence of IH: out of the 62 patients who developed an IH, 25 of them appeared in the midline component of the incision (40.3%), 13 appeared in the central part of the incision (21%), 15 formed in the transverse aspect of the incision close to the midline (24.2%), and the remaining 9 formed on the right edge of the wound (14.5%) (28). These results further support that the midline component of these incisions has the highest risk of developing an IH.

Memba et al. performed a systematic review of 8 studies about IH prevention in open HPB surgery. 6 of them were retrospective and the remaining 2 were prospective cohorts. Most of them shared the primary variable of IH incidence, while also evaluating risk factors for IH formation in this group of patients. They found a pooled IH incidence that ranged from 7.7% to 38.8%. They also described risk factors according to statistical significance and found that a high BMI, surgical site infection, ascites, Mercedes or reversed T incisions, and previous IH were related to a higher risk of developing an IH (29).

There is no doubt that incisional hernia negatively impacts the quality of life of the patient. The abdominal bulge, pain, and discomfort limits daily activities, and complications like incarceration and obstruction may occur. Also, symptomatic incisional hernias have an indication of surgical repair, which increases the costs of healthcare, taking also in consideration the morbidity associated with incisional hernia repair. Therefore, a strategy to prevent this from happening is important, with an emphasis on a proper abdominal wall closure technique tailored to each patient undergoing surgery.

### Risk Factors

The most important moment to identify who is at risk to develop an IH is during the preoperative consultation. Several risk factors like BMI >26.0 kg/m^2^, a height >167.5 cm, diabetes mellitus, malnutrition, smoking, and anemia have been correlated positively with IH formation. Correction of these factors plays a vital role to prevent IH formation. Chen-Xu et al. also described a positive correlation between IH formation and perirenal fat thickness >14.7 mm, and subcutaneous fat >23.3 mm. Both factors further support the concept that a higher BMI and obesity have an important impact on a higher incidence of IH (17). A more recent study published by Nagaoka et al. identified the presence of intramuscular adipose tissue in patients who underwent hepatic resection as a significant risk factor for IH formation after surgery, with up to 20% of the patients developing an IH at 3 years follow-up (30). One of the most detailed descriptions of risk factors associated with a higher IH incidence in HPB surgery is published by Memba et al. They collected most of the data available on this topic and found that a high BMI was the most mentioned factor in most studies that also had a positive correlation with IH formation. Secondary to BMI came surgical site infection and the presence of ascites/cirrhosis. And finally, the incision type, being the Mercedes and the reverse-T incisions the most related to IH appearance. Many other risk factors were described with low association with IH incidence, such as previous hernia surgery, running versus mass suture closure of the abdominal wall, preoperative chemotherapy, superficial wound dehiscence, subcutaneous and perirenal fat thickness, and malignancy, among others (29).

## Closing the Incisions

Adequate closure in HBP surgery is essential to ensure wound healing and prevent IH formation. Appropriate identification of sheaths and muscle layers that are closed adequately along the incisions can be considered a must. When closing incisions than extend outside the lateral border of the posterior rectus sheath, we can make 3 main types of closure: a mass closure can be performed taking together 3 layers with the same bites, a 2 layer that takes a first layer with TA and IOM and a second layer with EOM or a three-layer closure taking independently the three muscle layers. This closure follows to the midline with a mass closure or a two-layer closure of the anterior and posterior rectus sheaths. We would recommend to use a multilayered closure in all circumstances. Additionally, the incorrect apposition of borders and the inappropriate reconstruction of the lateral border of the posterior rectus sheath where the IOM divides into the anterior and posterior lamella might also contribute to inadequate wound healing. In order to avoid this mistake, we recommend to observe the myofascial limits of the EO e IE muscle to provide an adequate orientation (Figure 1).

There is a discussion about whether a layered closure is better than a mass closure. Zhang et al. published a prospective study where they compared patients undergoing liver resection that were closed *via* a mass continuous suture vs. a layered interrupted suture. They found no differences regarding IH formation, but did describe a longer closure time for interrupted layered suture (31). A recent trial has shown a significant reduction of SSI using layer closure (32). Long-term results have not yet been published comparing these methods of closure. Several studies and guidelines have published recommendations to reduce the risk of IH when closing incisions, mostly directed to midline wounds, but that can be extrapolated to lateral and hybrid incisions. European Hernia Society guidelines recommend a continuous small-bites suture technique with a slowly absorbable suture for closure (Tissue bites of 5–9 mm from the wound edges limited to aponeurosis only, with stitches placed 5 mm apart from one another in a continuous suturing technique, using a 2–0 size thread). This technique provides a low-tension closure that guarantees sufficient tissue perfusion for proper wound healing. The small-bites technique also implies a suture length to wound length ratio of 4:1 (33). This may have some caveats when addressing transverse or hybrid wounds, as there are more planes to take into consideration when closing the incision. Also, surgeons must take into consideration that using small bites outside the linea semilunaris may be difficult due to the lack of proper aponeurosis, since the 3 lateral muscles are covered by a weak fascia. A study that retrospectively compared conventional suture with small bites failed to demonstrate a statistically significant difference in the incisional hernia rate (34). Davey et al analyzed various studies addressing this topic, one of them being the INLINE meta-analysis, that suggests that a running suture with a slowly absorbable material has lower IH incidence in midline laparotomies. The INSECT trial however showed no difference between suture types. The pooled data favored the use of slowly absorbable or non-absorbable with a continuous suture to decrease IH rates (23). Memba et al also reviewed available literature addressing closure methods in HPB surgery, one if them is a Cochrane review from 2017. It showed that the quality of evidence available is low and could not determine what was the best type of suture or closure technique in HPB surgery. They concluded that studies tend to lean towards using small bites with a running suture for fascial closure, as this has demonstrated benefits in reducing IH in midline laparotomies (29). This could be extrapolated to subcostal incisions but requires studies to generate evidence and recommendations. Again, the literature at out disposal is small, no comparative studies exist, and the ones published are very heterogenous.

Numerous reports document the use of a prophylactic mesh to prevent incisional hernia formation. Although it is a topic that not all surgeons agree on, there is literature that supports their use in patients at high risk of IH formation after midline incisions. The EHS has recently published their updated guidelines for closure of abdominal wall incisions and stated that mesh augmentation after suture closure of a midline incision in elective surgery can be considered to reduce IH formation when compared to primary suture closure only, without any significant increase of surgical site infection (33). Nevertheless, the quality of evidence is low, with a weak strength of recommendation. Also, there are no RCT studies so far that compare the use of prophylactic mesh vs. primary suture closure in not midline incisions.

Our group performed a comparative cohort study on the use of prophylactic meshes to prevent IH in bilateral subcostal laparotomies (6). We compared 57 patients who retrospectively were closed with primary suture only, with 58 patients in which a prophylactic mesh was used when closing the laparotomy. The method of closure was the same for both groups, using a standard 2-layer protocol with a running slowly absorbable monofilament suture of Poly-4 Hydroxybutyrate, in a 4:1 ratio, with stitches spaced 1 cm from each other and 1 cm from the wound edge. The first layer included the closing of the IOM, TA, and the posterior rectus sheath medially. The second layer encompassed the closing of the EOM, its fascia, and the anterior rectus sheath. The mesh was placed in the space between the internal and external oblique muscles, and when extending medially it was placed retromuscular over the posterior rectus sheath. At 24 months, IH incidence was lower in the mesh group than in the control group (1.72% vs. 17.54%), with no statistical difference in morbidity and mortality. This means that mesh implantation can be safely placed as a prophylactic measure to prevent IH in subcostal incisions often used in HBP surgery. Another study that supported the use of mesh as prevention comes also from Spain. This study retrospectively compared a cohort of patients undergoing emergency subcostal incisions with suture closure vs. a similar cohort with an onlay mesh reinforcement (35). They also found a significant difference in incisional hernia rate between groups: 3.8% in the mesh group vs. 19.1% in the suture group. Interestingly, there was no difference in wound morbidity between groups. RCTs are necessary to offer more evidence on the use of mesh in HPB surgery.

## Summary

The choice of incision type for open HPB surgery is usually a straightforward decision based on the target organ, the type of patient, the proposed surgery, and the surgeon’s preference. Non-midline or oblique incisions are most commonly used, as they can provide adequate exposure and are associated with less risk of incisional hernia formation (2). However, subcostal or hybrid incisions have drawbacks that have not been fully studied and could present difficulties when performing or deciding how to close the abdomen. It is necessary to know the anatomy of the abdominal wall in this region, as the overlapping muscles and the presence of the neurovascular supply are at risk of injury (11, 12). We were able to identify in the literature a few noteworthy points to take into consideration when approaching these incisions: preventing vertical extension of a transverse or oblique incision, leaving enough fascia to perform a proper closure, avoid too lateral extension to avoid injury to intercostal nerves (2, 11, 19), and the application of the principles of abdominal closure of midline (small bites, mesh prophylaxis) to these lateral wounds. By accounting for these variables, we may decrease the risk of IH, and also may have a positive impact on reducing bulging incidence (2). However, there are still many other questions that need to be answered in randomized clinical trials (Table 1): which incision is better, how can we adequately do these incisions, how is the best way to close.

**TABLE 1:** Future research topics for incisional hernia in HPB surgery.

Potential areas of research	
Type of incision	Extended vs. midline augmentation
	Subcostal vs. transverse
Closure of the incision	Layer vs. Mass
	Small bites vs. large bites
	Absorbable vs. non-absorbable vs. antibiotic coating
	Mesh augmentation
Patient Reported Outcomes	Incidence of incisional hernia
	Quality of life reported outcomes
